# CIB4 is essential for the haploid phase of spermatogenesis in mice^†^

**DOI:** 10.1093/biolre/ioaa059

**Published:** 2020-04-25

**Authors:** Zoulan Xu, Haruhiko Miyata, Yuki Kaneda, Julio M Castaneda, Yonggang Lu, Akane Morohoshi, Zhifeng Yu, Martin M Matzuk, Masahito Ikawa

**Affiliations:** 1 Department of Experimental Genome Research, Research Institute for Microbial Diseases, Osaka University, Suita, Osaka, Japan; 2 Graduate School of Pharmaceutical Sciences, Osaka University, Suita, Osaka, Japan; 3 Graduate School of Medicine, Osaka University, Suita, Osaka, Japan; 4 Center for Drug Discovery, Baylor College of Medicine, Houston, TX, USA; 5 Department of Pathology and Immunology, Baylor College of Medicine, Houston, TX, USA; 6 The Institute of Medical Science, The University of Tokyo, Minato-ku, Tokyo, Japan

**Keywords:** male infertility, sperm, spermatogenesis, testis

## Abstract

Spermatogenesis is a complex developmental process that involves the proliferation of diploid cells, meiotic division, and haploid differentiation. Many genes are shown to be essential for male fertility using knockout (KO) mice; however, there still remain genes to be analyzed to elucidate their molecular mechanism and their roles in spermatogenesis. Calcium- and integrin-binding protein 1 (CIB1) is a ubiquitously expressed protein that possesses three paralogs: CIB2, CIB3, and CIB4. It is reported that *Cib1* KO male mice are sterile due to impaired haploid differentiation. In this study, we discovered that *Cib4* is expressed strongly in mouse and human testis and begins expression during the haploid phase of spermatogenesis in mice. To analyze the function of CIB4 in vivo, we generated *Cib4* KO mice using the CRISPR/Cas9 system. *Cib4* KO male mice are sterile due to impaired haploid differentiation, phenocopying *Cib1* KO male mice. Spermatogenic cells isolated from seminiferous tubules demonstrate an essential function of CIB4 in the formation of the apical region of the sperm head. Further analysis of CIB4 function may shed light on the etiology of male infertility caused by spermatogenesis defects, and CIB4 could be a target for male contraceptives because of its dominant expression in the testis.

## Teaser

*Cib4* mutant mice exhibit male infertility due to impaired spermiogenesis.

## Introduction

Infertility is a serious health problem, affecting 15% of reproductive-aged couples worldwide, and male factors are involved in nearly 50% of these cases [1–3]. Male infertility is a complex multifactorial and pathological condition from the complete absence of spermatozoa in the testes to distinct alterations of sperm quality, 60% of which can be contributed by genetic factors [4–6]. Most cases contributing to male fertility are azoospermia that exhibits severe impairment in spermatogenesis [4, 5, 7].

Spermatogenesis is a complex process that involves cell proliferation, meiosis, and haploid differentiation. During spermatogenesis in the seminiferous tubules of the mammalian testis, diploid spermatogonia differentiate into primary spermatocytes. Following two consecutive meiotic divisions, haploid spermatids are produced. Spermatids then undergo marked morphological changes from an initial round shape to an elongated shape, eventually leading to the formation of spermatozoa [8, 9]. This transformation process during spermatogenesis is called spermiogenesis [10]. Mouse spermatogenesis has been defined morphologically and is divided into 12 stages. Although several genes are reported to be involved in spermatogenesis, all of the genes involved and the mechanisms that regulate this process are still unclear.

The calcium- and integrin-binding (CIB) family contains four members, *Cib1* to *Cib4,* which is characterized by multi-EF hand motifs [11, 12]. CIBs were first identified as intracellular binding partners of an α-integrin cytoplasmic domain [13], but subsequent research has revealed that CIBs can interact with not only integrin but also many other proteins [14–16]. Within the CIB family, CIB1 and CIB2 have been studied most extensively. Both genes are expressed ubiquitously, including in cochlear hair cells, but only CIB2 deletion impacts hearing function in mice [17–19]. CIB2 binds to the components of the hair cell mechanotransduction complex, TMC1 and TMC2, and regulates the function of the mechanotransducer channels [19]. CIB1 plays diverse roles in calcium signaling and cell migration, adhesion, proliferation, and survival in many cell types [20]. To analyze its function in vivo, *Cib1* was deleted in mice and KO males exhibit infertility due to impaired haploid differentiation of spermatogenesis [21]. The previous study indicated that *Cib1* is expressed in both somatic and germ cells throughout spermatogenesis, and it is unclear how CIB1 plays critical roles only in the haploid phase of spermatogenesis [21].

In contrast to CIB1 and CIB2, the functions of CIB3 or CIB4 remain unclear. It has been shown that *Cib4* is expressed specifically in the testis in sheep [22, 23], suggesting that CIB4 may play a role in male fertility. In this study, we mutated *Cib4* using the CRISPR/Cas9 system and analyzed its function in mice.

## Materials and methods

### Ethics statement

All animal experiments were approved by the Animal Care and Use Committee of the Research Institute for Microbial Diseases, Osaka University.

### Animals

Mice were purchased from CLEA Japan (Tokyo, Japan) or Japan SLC (Shizuoka, Japan).

### Generation of *Cib4* KO mice using the CRISPR/Cas9 system

Knockout (KO) mice were generated as described previously [24]. gRNA with fewer off-target sites that are close to the start codon was designed by the online source CRISPRdirect [25]. Plasmids expressing hCas9 and gRNA were prepared by ligating oligonucleotides into the pX330 [26, 27]. The gRNA target sequence was 5′-TTTAAGGTACCAGATGCAGT-3′. Superovulated B6D2F1 female mice were mated with B6D2F1 males and fertilized eggs were collected from the oviduct. *Cib4* was mutated by injecting the pX330 plasmids into the pronuclei of fertilized eggs. Injected embryos were cultured in KSOM medium [28] to the two-cell stage and transplanted into the oviducts of pseudopregnant Institute of Cancer Research (ICR) females at 0.5 days after mating with vasectomized males. The *Cib4* mutant F0 mice were identified by genomic PCR using primers listed in Supplementary Table S1. The DNA sequence of the mutant alleles was further confirmed by Sanger sequencing of the PCR product. After genotype validation, F0 mice underwent serial mating to generate homozygous mutant offspring.

### Reverse transcription polymerase chain reaction and isolation of RNA

Mouse multitissues and 1- to 5-week-old testes samples were obtained from C57BL/6N male mice. Human multitissues were obtained from the Human Tissue Acquisition and Pathology core service (Baylor college of Medicine, Houstan, TX). Informed consent of these human tissues was obtained. RNA samples were isolated and purified using TRIzol (Thermo Fisher Scientific, MA). RNA was reverse transcribed to cDNA using SuperScript III or SuperScript IV First-Strand Synthesis System (Thermo Fisher Scientific). PCR was performed using primers that are listed in Supplementary Table S1.

### In silico data analysis

Single-cell transcriptome data in the mouse and human testis [29] were obtained. CIB family expression in those cells was analyzed using Loupe Cell Browser 3.3.1 (10X Genomics, Pleasanton, CA).

### Mating test

Sexually mature KO male mice or control male mice were caged with two 8-week-old B6D2F1 female mice each for 2 months, and plugs were checked every morning. The number of pups was counted on the day of birth.

### Histology analysis

Periodic acid–Schiff (PAS) staining of sections were performed as previously described [30]. Testes or epididymis were fixed in Bouin solution (Polysciences, Inc., Warrington, PA) and were processed for paraffin embedding. Paraffin sections were cut at a thickness of 5 μm on a Microm HM325 microtome (Microm, Walldorf, Germany) and stained with 1% periodic acid (Nacalai Tesque, Kyoto, Japan) and Schiff reagent (FUJIFILM WakoPure Chemical, Osaka, Japan) followed by counterstaining with Mayer hematoxylin solution (FUJIFILM WakoPure Chemical).

**Figure 1:**
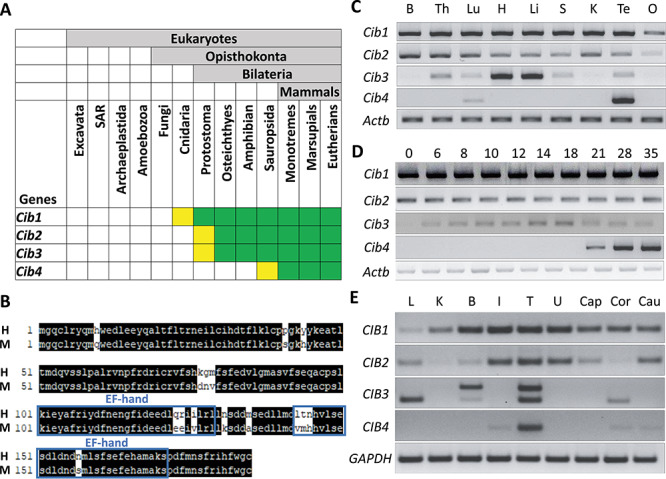
CIB4 is testis-enriched in mice and humans. (A) Conservation of CIB family. Green indicates presence of CIB in most species within taxon. Yellow indicates loss in several species within taxon. (B) Alignment of amino acid sequences between mice and human. Matched sequences are highlighted in black, and EF-hands are indicated in blue boxes. H: human CIB4, M: mouse CIB4. (C) Expression of mouse *Cib* family members was analyzed by RT-PCR using various organs. *Actb* was used as an RNA expression control. B: brain, Th: thymus, Lu: lung, H: heart, Li: liver, S: spleen, K: kidney, Te: testis, O: ovary. (D) Expression of mouse *Cib* family members was analyzed by RT-PCR using various postnatal testes. *Actb* was used as an RNA expression control. (E) Expression of the human *CIB* family members was analyzed by RT-PCR using various organs. Two bands were observed for *CIB3* likely due to the existence of variants. *GAPDH* was used as an RNA expression control. L: liver, K: kidney, B: brain, I: intestine, T: testis, U: uterus, Cap: caput epididymis, Cor: corpus epididymis, Cau: cauda epididymis.

### Immunofluorescence

Testis cross-sections were obtained as described above. The sections were deparaffinized, rehydrated, and permeabilized with 0.1% Triton X-100 in phosphate-buffered saline (PBS). The slides were then blocked with 5% bovine serum albumin (BSA) and 10% goat serum for 120 min at room temperature and incubated with primary antibody overnight at 4 °C. Samples were incubated with secondary antibody at room temperature for 90 min and stained with Hoechst 33342 (H3570, Thermo Fisher Scientific) for 15 min. The samples were mounted with Immu-Mount (9990402, Thermo Fisher Scientific) and observed with a BX-53 microscope (Olympus, Tokyo, Japan). The antibodies used were anti-acetylated tubulin (#T7451, Sigma-Aldrich, St. Louis, MO) for the primary antibody and Alexa Fluor 488 conjugated secondary antibody (Thermo Fisher Scientific).

Immunofluorescence for the manchette was conducted as described previously [24]. Germ cells, including spermatids, were obtained from the seminiferous tubules, dried in a 37 °C incubator, and fixed with 4% paraformaldehyde in PBS for 15 min. After permeabilization with 0.1% Triton X-100 for 15 min, the samples were blocked with 5% BSA and 10% goat serum diluted in PBS for 60 min at room temperature. The samples were incubated with the primary antibody overnight at 4 °C and secondary antibody for 60 min at room temperature. Samples were stained with Hoechst 33342 (H3570, Thermo Fisher Scientific) for 15 min and observed with a BX-53 microscope (Olympus). Antibodies used were anti-α-tubulin (B-5-1-2, #T5168, Sigma-Aldrich) for primary antibody and Alexa Fluor 488 conjugated secondary antibody (Thermo Fisher Scientific).

### Construction of plasmids

cDNAs of *Cib1* and *Cib4* were amplified from C57BL/6N mouse testis and cloned into FLAG-tagged (C-terminus) or PA-tagged (C-terminus) pCAG vectors that contain the CAG promoter and a rabbit globin poly(A) signal [31]. The primers used are listed in Supplementary Table S1. FLAG-tagged *Drc7* vector and PA-tagged *Drc3* vector were described previously [30].

### Cell culture and transfection

Culture of HEK293T, transfection of plasmid DNAs, and immunoprecipitation were performed as described previously [30]. Rat anti-PA antibody (#012–25863, FUJIFILM Wako Pure Chemical) was used for immunoprecipitation.

**Figure 2:**
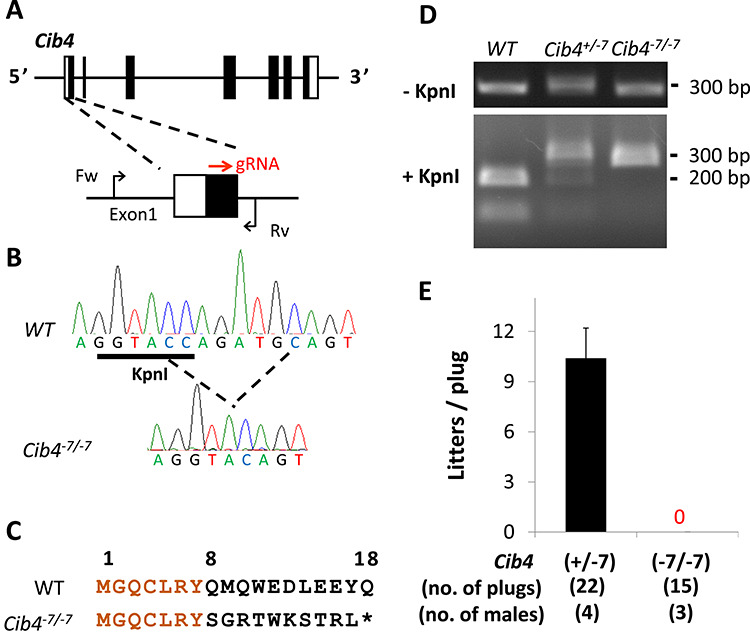
Generation of *Cib4* KO mice using the CRISPR/Cas9 system. (A) CRISPR/Cas9 targeting scheme. gRNA was targeted to Exon1. Primers (Fw, Rv) used for genotyping are shown. (B) Wave pattern sequence of *Cib4* confirming the targeted allele. (C) Amino acid sequence of CIB4. Asterisk indicates stop codon. (D) Genotyping of *Cib4^–7/−7^* mice using KpnI. (E) Fertility of *Cib4^–7/−7^* male mice.

### Immunoblot analysis

Immunoblot analysis was performed as described previously [32]. Proteins were separated by sodium dodecyl sulfate-and polyacrylamide gel electrophoresis followed by western blotting. The blots were blocked with 10% skim milk and incubated with primary antibodies overnight at 4 °C. Primary antibodies used were rat anti-PA antibody, 1:1000 (#012–25863, FUJIFILM Wako Pure Chemical); rabbit anti-FLAG antibody, 1:1000 (#PM020, Medical & Biological Laboratories, Aichi, Japan); and mouse anti-α-tubulin antibody, 1:1000 (B-5-1-2, #T5168, Sigma-Aldrich). The blots were then incubated with horseradish peroxidase-conjugated secondary antibodies for 2 h at room temperature (Jackson ImmunoResearch, West Grove, PA). Immunoreactive proteins were detected by an ECL western blotting detection kit (GE Healthcare, Little Chalfont, UK).

### Statistical analysis

Statistical analyses were carried out using the two-tailed Student *t*-test. Differences were considered significant at *P* < 0.05 (^*^) or highly significant at *P* < 0.01 (^*^^*^). Error bars are standard deviation.

## Results

### CIB4 is a testis-enriched protein

CIB4 belongs to the CIB protein family, composed of four members, CIB1, CIB2, CIB3, and CIB4. This family is characterized by several EF-hand domains and is well conserved in mammals (Figure 1A and B). To determine the expression pattern of *Cib* family genes in mice, we performed reverse transcription polymerase chain reaction (RT-PCR) using RNAs obtained from multiple mouse tissues (Figure 1C). *Cib1* and *Cib2* were expressed ubiquitously, while *Cib3* is expressed strongly in the heart and liver. *Cib4* is expressed predominantly in the testis with weak expression detected in the lung. We then conducted RT-PCR using RNAs obtained from postnatal mouse testes (Figure 1D). *Cib1* starts to express on day 0, which is consistent with the previous study showing *Cib1* expression in Sertoli cells and spermatogonia [21]. *Cib2* and *Cib3* start to express on day 0 and day 6, respectively. In contrast, *Cib4* starts to express on day 21 when spermiogenesis begins.

RT-PCR was also performed using RNAs obtained from multiple human tissues (Figure 1E). Consistent with the expression pattern in mice, *CIB1* and *CIB2* are expressed ubiquitously. In contrast, *CIB3* is expressed in liver, brain, and testis; *CIB4* is expressed predominantly in testis. The expression of *Cib4* in the haploid phase of spermatogenesis is confirmed in both mice and humans using an in silico approach by examining an expression database (Supplementary Figure S1A and B). These data suggest that CIB4 may play roles in postmeiotic stages of spermatogenesis.

**Figure 3:**
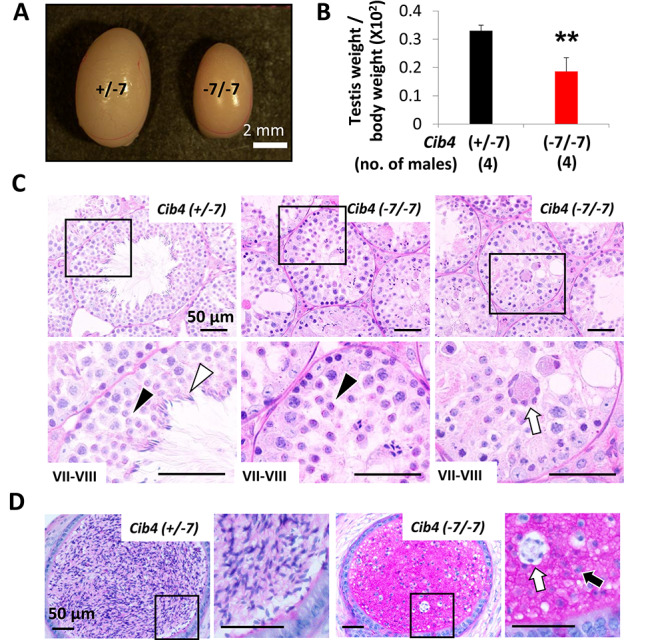
*Cib4^–7/−7^* mice are infertile with small testis size. (A) Testes of *Cib4^+/−7^* and *Cib4^–7/−7^* mice. Scale bar: 2 mm. (B) The average testis weight (one testis) per body weight of *Cib4^+/−7^* and *Cib4^–7/−7^* mice. ^*^^*^P < 0.01, Student *t*-test, error bars represent SD. (C) PAS staining of testes cross-sections. In stage VII–VIII seminiferous tubules, normal round spermatids (black arrowheads) were observed; however, elongated spermatids (white arrowhead) were hardly found in *Cib4^–7/−7^* testis. Multinucleated cells (white arrow) were frequently detected in the lumen of seminiferous tubules. Black rectangles indicate magnified regions. (D) PAS staining of cauda epididymis. Round spermatid-like cells (black arrow) and multinucleated cells (white arrow) were observed in *Cib4^–7/−7^* epididymis. Black rectangles indicate magnified regions. Twelve-week-old mice were used for A–D.

### Generation of *Cib4* KO mice using the CRISPR/Cas9 system and demonstration of infertility

To uncover the functions of *Cib4* in vivo, we generated KO mice using the CRISPR/Cas9 system. The mouse *Cib4* gene contains seven exons encoding 185 amino acids, and the gRNA was designed to target exon 1 (Figure 2A). Obtained pups possess a 7 bp-deletion (Figure 2B), which resulted in a frameshift mutation (Q8S) and introduction of nine new amino acids, and a premature stop codon (Figure 2C). Because this deletion disrupts the KpnI restriction enzyme site, genotypes can be determined by digesting the PCR product with the KpnI enzyme (Figure 2D). *Cib4^–7/−7^* mice were obtained by subsequent matings and did not exhibit overt abnormalities.

*Cib4^+/−7^*or *Cib4^–7/−7^* male mice were housed with two wild-type (WT) females to check their fecundity. Even though 15 copulatory plugs were observed, no pups were obtained from *Cib4^–7/−7^* mice (Figure 2E), indicating that *Cib4* is essential for male fertility.

### Loss of CIB4 results in impaired spermatogenesis in male mice

To investigate the cause of male infertility, we observed testes of *Cib4^+/−7^*and *Cib4^–7/−7^* adult male mice (Figure 3A) and found that the average testis weight of *Cib4^–7/−7^* mice is smaller than that of *Cib4^+/−7^* mice (Figure 3B). These results suggest that *Cib4^–7/−7^* male mice are sterile due to impaired spermatogenesis.

**Figure 4:**
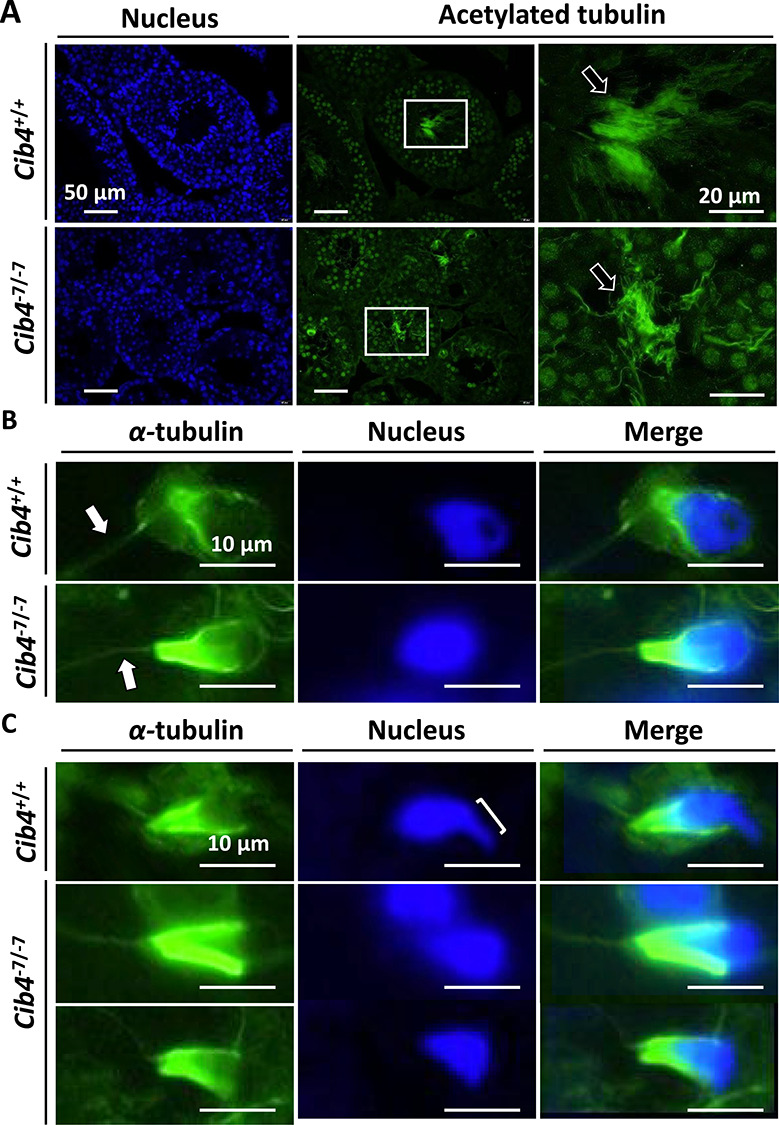
Tail and manchette formation in *Cib4^–7/−7^* testis. (A) Immunofluorescence analysis of tail formation of spermatids in *Cib4^–7/−7^* testis. Nucleus (blue), acetylated-tubulin (green). Arrows indicate tail. White rectangles indicate magnified regions. (B) Immunofluorescence analysis of the manchette of round spermatids. α-tubulin (green) stains both tails and manchettes. Arrows indicate the tail of one spermatid. (C) Immunofluorescence analysis of the manchette of elongating spermatids. Bracket indicates formation of the apical head, which was not observed in *Cib4^–7/−7^* mice.

**Figure 5:**
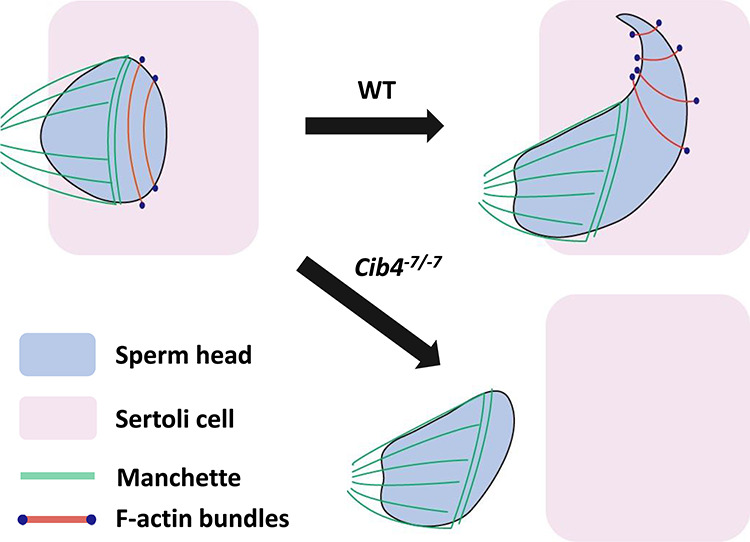
Hypothetical schematic of sperm head elongation in WT and *Cib4^–7/−7^* mice. During the elongation of sperm heads, manchette compresses the caudal region both in WT and *Cib4^–7/−7^* mice. In the later stage of elongating spermatids, hook shapes are formed with F-actin bundles in the apical region of the heads in WT mice; however, no hook shapes were formed in *Cib4^–7/−7^* mice.

To define the stage of spermatogenesis that is impaired, we conducted a histological analysis of testes. We found that spermatogonia and primary spermatocytes were comparable at stages VII–VIII between *Cib4^+/−7^* and *Cib4^–7/−7^* testis (Figure 3C). Further, round spermatids were detected at this stage. However, the layers of spermatogenic cells in the seminiferous tubules were disorganized, and elongated spermatids were rarely observed (Figure 3C). Further, large multinucleated cells containing numerous spermatids were frequently observed in the lumen of seminiferous tubules in the *Cib4^–7/−7^* testis (Figure 3C)*.* At stages IX–X, the round spermatids start to elongate, which is characterized by progressive elongation and condensation of the nucleus. However, elongated spermatids were rarely detected in this stage in *Cib4^–7/−7^* testis (Supplementary Figure S2).

Consistent with the observation of small testis in *Cib4^–7/−7^* mice, impaired spermatogenesis at the postmeiotic stage was confirmed by observing the first wave of spermatogenesis (Supplementary Figure S3A). Round spermatids were comparable between *Cib4^+/−7^* and *Cib4^–7/−7^* testes at postnatal day 21 (Supplementary Figure S3A), while impaired spermatid elongation was observed in the *Cib4^–7/−7^* testis at postnatal day 28 (Supplementary Figure S3B). Consistent with these results, no elongated mature spermatozoa were detected in the cauda epididymis of *Cib4^–7/−7^* mice and instead round spermatid-like cells were observed (Figure 3D). These results indicate that elongation of spermatids is impaired in *Cib4^–7/−7^* mice, phenocopying *Cib1* KO mice [21]. Multinucleated cells containing numerous spermatids were frequently observed in *Cib1* KO mice as well.

### Immunostaining of testis sections and manchette staining reveal a *Cib4* KO sperm head defect

To further analyze the impaired haploid differentiation, we performed immunohistochemical staining for acetylated tubulin to observe the formation of sperm flagella. As indicated in Figure 4A, acetylated tubulin signals were observed in both *Cib4^+/−7^* and *Cib4^–7/−7^* testes, suggesting that flagella can form without CIB4.

We then collected spermatogenic cells from seminiferous tubules and stained for α-tubulin to observe flagella and the manchette, a transient microtubule-based structure that is essential for the formation of flagella and caudal region of sperm heads [33, 34]. Consistent with Figure 4A, elongation of flagella was observed in germ cells from *Cib4^–7/−7^* mice (Figure 4B). Further, the formation of the manchette was comparable between *Cib4^+/−7^* and *Cib4^–7/−7^* mice (Figure 4B). We then observed elongating spermatids and found that the manchette could compress the caudal region of the sperm head in both *Cib4^+/−7^* and *Cib4^–7/−7^* mice (Figure 4C). In contrast, hook shapes of the apical region of the sperm heads were not formed in the *Cib4^–7/−7^* spermatids. These results reveal that CIB4 is essential for the formation of the apical region of the sperm heads (Figure 5).

### Interaction of CIB1 and CIB4 is not detected in HEK293T cells

Because *Cib4^–7/−7^* mice phenocopy *Cib1* KO mice and both *Cib1* and *Cib4* are expressed in germ cells, we analyzed the interaction of CIB1 and CIB4 by co-expression in HEK293T cells. While interaction between DRC3 and DRC4, two known interactors [30], is detected, CIB4-FLAG was not pulled down with CIB1-PA (Supplementary Figure S4), suggesting that CIB4 does not interact directly with CIB1.

## Discussion

In this study, we showed that *Cib4* starts to express at the haploid phase of spermatogenesis. Further, we revealed that *Cib4^–7/−7^* male mice are sterile due to spermatogenetic defects. Although *Cib4^–7/−7^* males exhibit normal sexual behavior, they had smaller testes and failed to produce the next generation. Impaired spermatogenesis results in the absence of elongated spermatozoa in the cauda epididymis of *Cib4^–7/−7^* mice.

Immunofluorescence analysis of spermatogenic cells with an α-tubulin antibody indicates that flagella can elongate, and the manchette can compress the caudal region of sperm heads in *Cib4^–7/−7^* mice (Figure 5). In contrast, hook shapes of the apical region of sperm heads were not formed. To form the apical region of sperm heads, spermatids should be surrounded by Sertoli cells, and the sperm nuclei should be compressed by bundles of actin filaments, called F-actin hoops, that are localized inside the Sertoli cells (Figure 5) [33, 34]. Because CIB4 is expressed in germ cells, but not in Sertoli cells, CIB4 may be involved in the interaction of germ cells and Sertoli cells rather than being directly involved in the formation of F-actin hoops in the Sertoli cells. This idea is supported by the observation of aggregated round spermatids that are released abnormally into the lumen of seminiferous tubules (Figure 3C) and round spermatid-like cells that are subsequently observed in the cauda epididymis (Figure 3D).

Our analysis of *Cib4^–7/−7^* mice indicates that their phenotypes are similar to those of *Cib1* KO mice. Considering that *Cib1* is expressed in germ cells as well as Sertoli cells, CIB1 and CIB4 may work together for the elongation of sperm heads in germ cells. In the mouse cochlear hair cells, the localization of CIB1 changes from the cell periphery to the apical cell center in the absence of CIB2 [17], suggesting that there may be interaction among CIB proteins. However, we cannot detect direct interaction between CIB1 and CIB4 (Supplementary Figure S4), indicating that CIB1 and CIB4 may not associate or associate indirectly. Alternatively, CIB1 may play more critical roles independently from CIB4 in Sertoli cells for the interaction between germ cells and Sertoli cells. Further analysis is necessary to understand the roles of CIB1 and CIB4 and their relationship in haploid differentiation.

In summary, we reveal that *Cib4* is essential for the haploid phase of spermatogenesis, especially for the formation of the apical region of sperm heads, and deletion of *Cib4* in mice causes absence of elongated spermatozoa in the cauda epididymis. Elucidating the function of *Cib4* in spermatogenesis may lead to better treatment of individuals with azoospermia. In addition, because *CIB4* is expressed strongly in human testis, CIB4 can be a good target for a nonhormonal male contraceptive that does not disrupt the diploid or meiotic phase of spermatogenesis. Considering that CIB4 is an intracellular protein, looking for small molecules that can pass through the blood-testis barrier and plasma membrane and inhibit CIB4 function may lead to the development of contraceptives. The crystal structure of CIB1 that was solved previously [35] may be useful to find small molecules that specifically bind to CIB4.

## Supplementary Material

Xu_et_al_Supplementary_Data_ioaa059
