# Supplementary material for: CIB4 is essential for the haploid phase of spermatogenesis in mice
Source: Biol Reprod. 2020 Apr 25;103(2):235–43. doi: 10.1093/biolre/ioaa059 (PMC7401386; doi:10.1093/biolre/ioaa059)
Supplement: Xu_et_al_Supplementary_Data_ioaa059 [file xu_et_al_supplementary_data_ioaa059.pdf]

Supplementary Figure S1

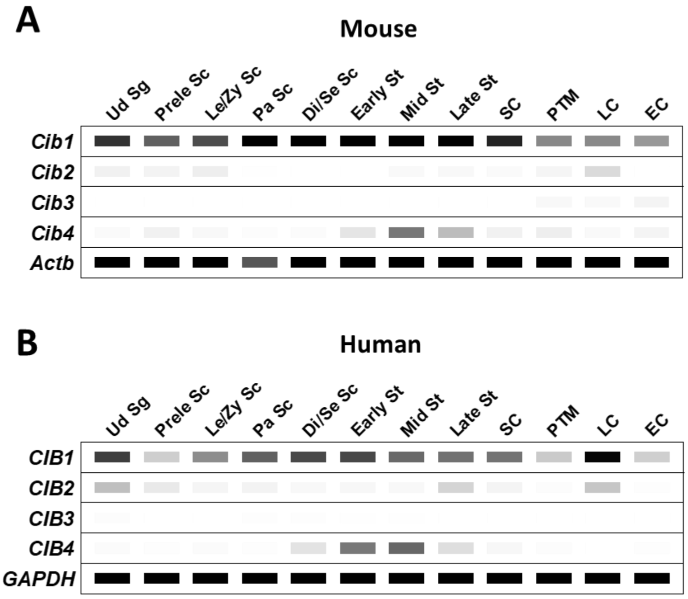

Supplementary Figure S1. Single-cell RNA-sequencing database depicting the expression of CIB family during mouse and human spermatogenesis.

A. Median-normalized levels of mRNA expression of the *Cib* family members during spermatogenesis in mice. *Actb* was used as an expression control. The level of mRNA expression in each cell type is indicated by the intensity of each band. White: median-normalized reads = 0; Black: median-normalized reads ≥ 4; Gray: 0 < median-normalized reads < 4. **B**. Median-normalized levels of mRNA expression of CIB family members during spermatogenesis in human. GAPDH was used as an expression control. The level of mRNA expression in each cell type is indicated by the intensity of each band. White: median-normalized reads = 0; Black: median-normalized reads ≥ 6; Gray: 0 < median-normalized reads < 6. Ud Sg, undifferentiated spermatogonia; Prele Sc, preleptotene spermatocytes; Le/Zy Sc, leptotene/zygotene spermatocytes; Pa Sc, pachytene spermatocytes; Di/Se Sc, diplotene/secondary spermatocytes; Early St, early round spermatids; Mid St, mid round spermatids; Late St, late round spermatids; SC, Sertoli cells; PTM, peritubular myoid cells; LC, Leydig cells; EC, endothelial cells.

Supplementary Figure S2

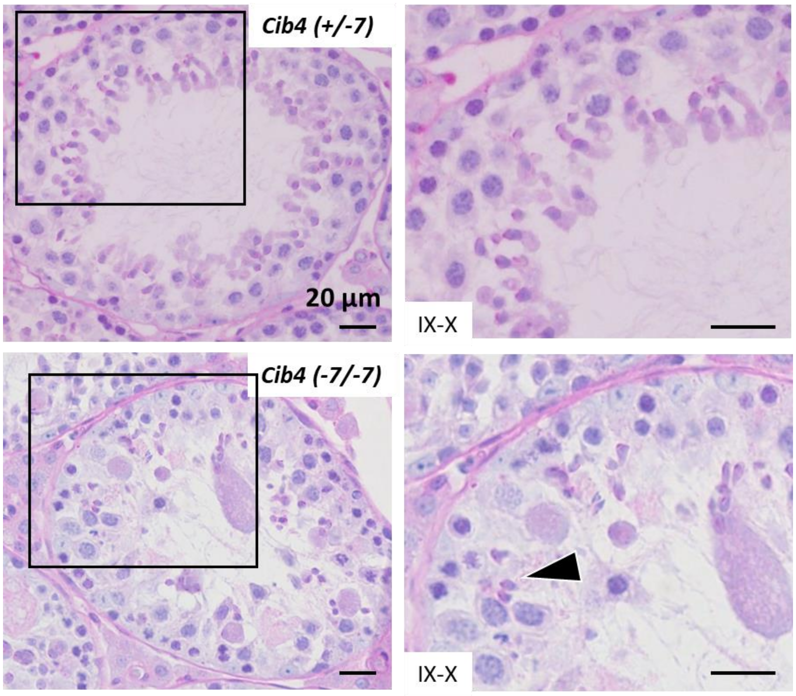

Supplementary Figure S2. PAS staining of stage IX-X seminiferous tubules.

In stage IX-X seminiferous tubules, there are fewer elongating spermatids (black arrow) in *Cib4*$^{-7/-7}$ testis. Black rectangles indicate magnified regions.

Supplementary Figure S3

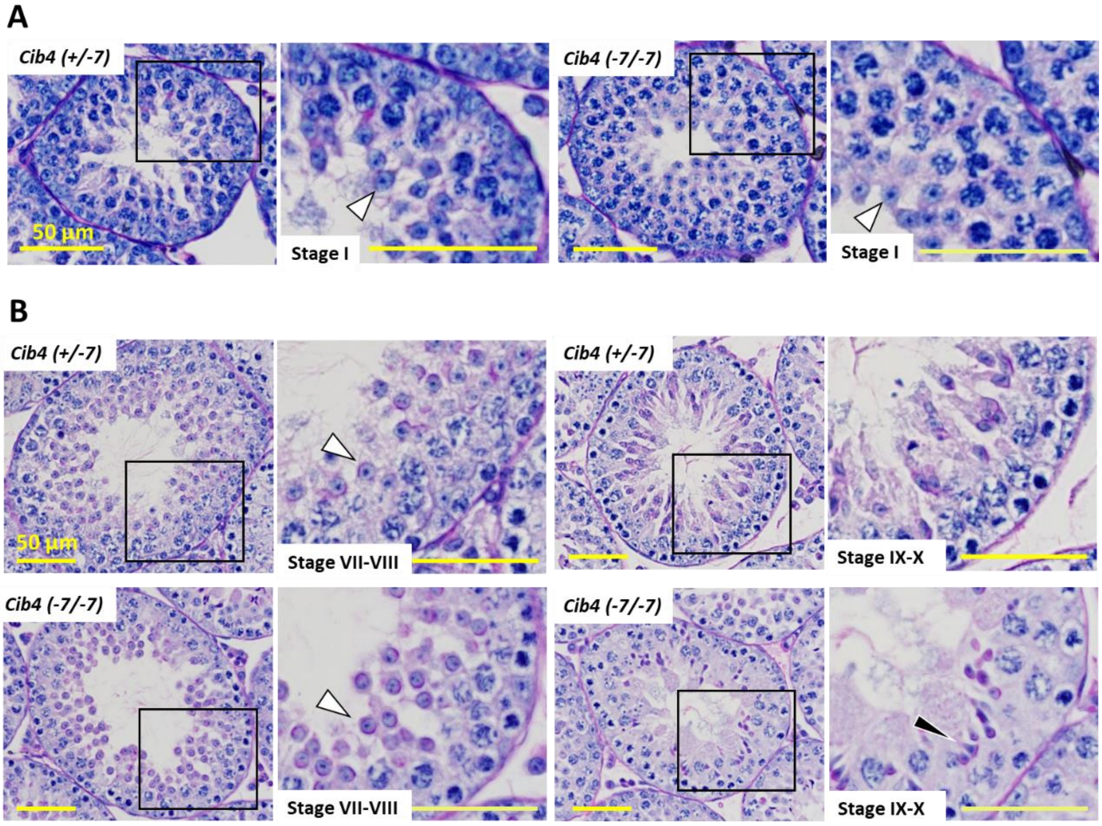

Supplementary Figure S3. First wave of spermatogenesis at postnatal day 21 and day 28. **A**. PAS staining of testes cross-sections at postnatal day 21 showed normal round spermatids (white arrowheads) in stage I seminiferous tubules of *Cib4*$^{+/-7}$and *Cib4*$^{-7/-7}$ mice. **B**. PAS staining of testes cross-sections at postnatal day 28 showed normal round spermatids (white arrowheads) in stage VII-VIII seminiferous tubules of *Cib4*$^{+/-7}$and *Cib4*$^{-7/-7}$ mice. In contrast, abnormally transformed spermatids (black arrowhead) were found in stage IX-X seminiferous tubules of *Cib4*$^{-7/-7}$ mice. Black rectangles indicate magnified regions.

Supplementary Figure S4

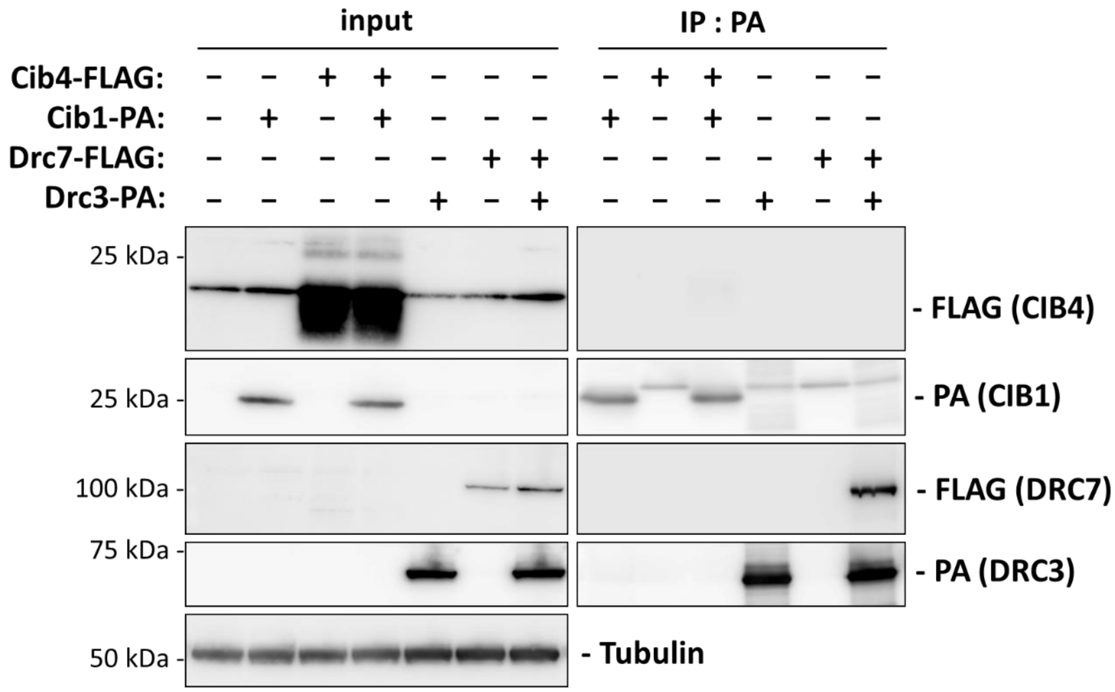

Supplementary Figure S4. Analysis of CIB1-CIB4 interaction.

Interaction of CIB1-PA and CIB4-FLAG was not detected. Interaction of DRC3-PA and DRC7-FLAG was confirmed as a positive control. α-Tubulin was used as a loading control.

Supplementary Table S1. Sequences of primers

Gene	Method	Species	Forward Primer	Reverse Primer
Cib1	RT	Mouse	ATGGGAGGTTCGGGCAGTCG	CAGGACAATCTTAAAGGAGC
Cib2	RT	Mouse	GACCATCTTCACTGAAGAGC	GATTCGAATGTGGAAGGTGC
Cib3	RT	Mouse	AGGCTGTTCTATCGATACC	GATGCGGATGTGGAAGGTGC
Cib4	RT	Mouse	AAGGTACCAGATGCAGTGGG	GCAGCCCCAGAAGTGAATCC
Actb	RT	Mouse	CATCCGTAAAGACCTCTATGCCAAC	ATGGAGCCACCGATCCACA
Cib4	Genotyping	Mouse	TGGAGGTGAGGGTTCTTAGC	GTTCTGTCCTGGATCTGTGACC
CIB1	RT	Human	CAGATTCTCAGCCTTCCAGAG	CAAAGTCAAAGATGCGGAAGG
CIB2	RT	Human	TCGAGGACATGATTGCCAAG	GTGTAAACCCCAGAGGCTG
CIB3	RT	Human	GCGACCTCAAGGCTTACTATG	CAGCACCTTCTCACATACCAG
CIB4	RT	Human	AGCCTGAAGATTGAGTATGCC	AACTCTGAGAAGGACAGCATG
GAPDH	RT	Human	AATCCCATCACCATCTTCCAG	ATGACCCTTTTGGCTCCC
Cib1	Cloning	Mouse	AAGGATCCGCCGCCATGGGAGGT TCGGGCAGTCG	AAGTCGACCAGGACAATCTTAA AGGAGC
Cib4	Cloning	Mouse	AAGGATCCGCCGCCATGGGGCA GTGTTTAAGG	AAGAATTCGCAGCCCCAGAAGT GAATCC
